# Monoclonal Annular Lichenoid Dermatitis of Youth as a New Entity: A Case Report and Review of the Literature

**DOI:** 10.3390/ijms27093990

**Published:** 2026-04-29

**Authors:** Olga Tockova, Violeta Hosta, Tanja Planinsek Rucigaj, Svjetlana Ponorac, Ira Kokovic, Eduardo Calonje, Bostjan Luzar

**Affiliations:** 1 Department of Dermatovenereology, University Medical Centre Ljubljana, 1000 Ljubljana, Slovenia; violeta.hosta@kclj.si (V.H.); t.rucigaj@gmail.com (T.P.R.); 2 Juventina Clinic, 1000 Ljubljana, Slovenia; svetlana.ponorac@gmail.com; 3 Department of Molecular Diagnostics, Institute of Oncology Ljubljana, 1000 Ljubljana, Slovenia; kokovic.ira@gmail.com; 4 St John’s Institute of Dermatology, South Wing, St Thomas’ Hospital, London SE1 7EP, UK; eduardo.calonje@gstt.nhs.uk; 5 Institute of Pathology, University of Ljubljana, 1000 Ljubljana, Slovenia; bostjan.luzar@mf.uni-lj.si; 6 Faculty of Medicine, University of Ljubljana, 1000 Ljubljana, Slovenia

**Keywords:** T-cell monoclonality, T-cell receptor rearrangement, annular lichenoid dermatosis, youth, cutaneous T-cell lymphoma, dermatopathology

## Abstract

Annular lichenoid dermatitis of youth (ALDY) is a rare lichenoid dermatosis characterized by distinctive clinical and histopathological features. Its etiopathogenesis remains poorly understood, and previously reported cases have consistently demonstrated polyclonal T-cell receptor (TCR) gene rearrangements. We report a patient with clinical, histopathological, and immunohistochemical findings consistent with ALDY in whom molecular analysis revealed monoclonal T-cell receptor rearrangement within the skin lesions. To our knowledge, this represents the first reported case of ALDY demonstrating T-cell monoclonality. This novel finding expands the current understanding of the molecular spectrum of ALDY and raises the possibility that cases with monoclonal T-cell rearrangement may represent a distinct clinicopathological variant. Based on our findings, we tentatively propose the term monoclonal annular lichenoid dermatitis of youth (MALDY) to describe this potential entity. Further studies are warranted to clarify its clinical significance and relationship to other cutaneous T-cell disorders.

## 1. Introduction

Annular lichenoid dermatitis of youth (ALDY) is an uncommon inflammatory skin disorder belonging to the group of lichenoid dermatoses and displaying characteristic clinical and histopathological features. The condition was initially reported in 2003 [1]. To date, only several dozen cases of ALDY have been reported [1,2,3,4] (Table 1).

It mostly affects pediatric and adolescent populations; however, adult cases have also been documented. Reported patient ages range from 2 to 79 years, with a mean age of 14.7 years and a median age of 10.5 years [1,2,4]. A slight male predominance has been observed, although the reason for this remains unclear [11,15,17,18]. Its etiopathogenesis remains poorly understood. Although various triggers, including infections, medications, tick bites, autoimmune conditions, and malignancies, have been proposed, these associations are not supported by consistent evidence and should be considered speculative [1,2,3].

From a clinical perspective, ALDY typically presents as single or multiple asymptomatic annular red-brown macules or patches with a mildly palpable erythematous rim and a hypopigmented central area [1,2,15,17,18]. The hyperpigmented border and small lichenoid papules may occasionally develop along the margins of the lesions [2,3]. Early lesions are, in fact, well-demarcated, round to oval erythematous macules that may enlarge centrifugally into annular patches [1,2]. As the disease progresses, these lesions evolve into fully developed plaques, which are typically round to oval, with a red-brown to violaceous, non-scaling, slightly raised border and a centrally hypopigmented area [1,2,3]. The lesions are generally non-indurated, with a smooth surface and soft consistency [4,11]. In later stages, the erythema gradually fades, leaving residual hyperpigmented rings surrounding non-atrophic areas [17,18]. The condition is typically asymptomatic; however, some patients may report mild pruritus, particularly during the early stages of lesion development. No significant systemic symptoms are usually present [1,2,3]. Despite increasing recognition, ALDY remains underreported and incompletely characterized, particularly regarding its clinical variability and potential triggers.

ALDY generally exhibits a chronic course. Although spontaneous regression can occur, recurrence or reappearance of lesions has also been reported [1,3,4]. Even though ALDY does not demonstrate specific predilection sites, lesions are most frequently observed on the trunk and often involve flexural regions, including the axillae, inframammary areas, periumbilical region, neck, and inguinal folds [2,3]. Currently, no standardized treatment strategy has been established and commonly used treatments such as topical corticosteroids and calcineurin inhibitors demonstrate variable effectiveness [2,3]. It may clinically resemble inflammatory morphea, mycosis fungoides (MF), annular erythema, or interstitial granulomatous dermatitis (Table 2).

Histopathologically, ALDY is characterized by a distinctive yet variable lichenoid tissue reaction pattern, with subtle differences depending on disease stage and individual cases [1]. Although traditionally considered a relatively uniform entity, the literature demonstrates a broader spectrum of histopathological presentations [1,2]. The classic pattern includes lichenoid interface dermatitis predominantly affecting the tips of rete ridges, irregular epidermal hyperplasia with alternating thinned and quadrangular rete ridges, a band-like lymphocytic infiltrate in the papillary dermis, and basal keratinocyte damage with vacuolar degeneration and apoptotic keratinocytes [1,2,7]. The preferential involvement of rete ridge tips, with relative sparing of the inter-rete epidermis, represents a rete ridge-targeted (localized interface) variant [2,7]. Histological features may evolve over time, with early lesions showing subtle interface changes, fully developed lesions demonstrating a pronounced lichenoid infiltrate and architectural epidermal alterations, and late lesions exhibiting epidermal flattening and reduced inflammation [1,2,3]. In some cases, a more diffuse interface dermatitis resembling lichen planus may be observed, while rare cases may display pseudolymphomatous or mycosis fungoides-like features, including dense infiltrates and epidermotropism, necessitating careful clinicopathological correlation [2,3,7]. Immunohistochemically, dermal infiltrates consist predominantly of CD2+, CD3+, CD4+, CD5+, and CD7+ lymphocytes, whereas most intraepidermal T cells are CD8+ [2].

To date, molecular analyses of T-cell receptor (TCR) gene rearrangement in ALDY have consistently demonstrated polyclonality, supporting its classification as a benign inflammatory condition [4,11,15].

Herein, we report a case of ALDY with monoclonal T-cell receptor rearrangement detected in skin lesions, representing, to our knowledge, the first such observation. This finding challenges the current understanding of ALDY and raises the possibility of a distinct clinicopathological variant.

The aim of this report is to describe this unusual molecular finding, further characterize the clinical and histopathological features of ALDY, and contribute additional data to better define this rare entity and its potential implications.

## 2. Case Report

**Figure 1:**
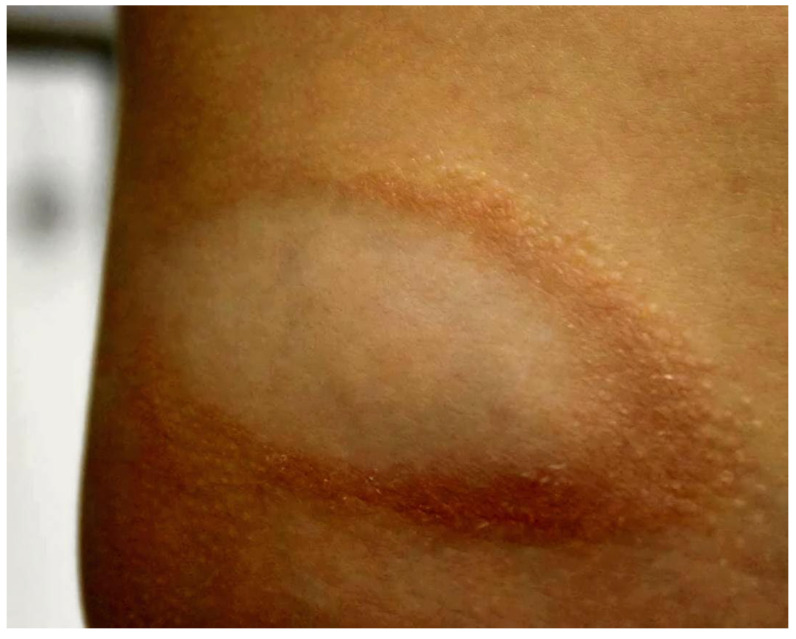
Red-brown annular plaque with non-atrophic, hypopigmented center and indurated border, on the right flank.

A 15-year-old boy presented to our department with a 3-year history of an asymptomatic red-brown annular lesion on the right flank (Figure 1). His medical history was notable for asthma, allergic rhinitis, and grass pollen allergy as type I hypersensitivity reactions; however, he was not receiving any medication at the time of presentation.

Dermatological examination revealed asymptomatic reddish-brown annular plaques measuring 4–6 cm, with a non-atrophic, hypopigmented center, indurated borders, and small lichenoid papules at the periphery, located on the right flank. At presentation, the patient was afebrile with no significant systemic symptoms.

To exclude other potential etiopathological factors contributing to the patient’s cutaneous manifestations, an extensive diagnostic workup was performed. Routine laboratory investigations were unremarkable. These included erythrocyte sedimentation rate, C-reactive protein, complete blood count with differential, serum creatinine, liver function tests, urinalysis, anti-tissue transglutaminase (tTG) antibodies, and anti-endomysial antibodies (EMA). Immunoserological evaluation, including Hep-2 antinuclear antibodies, an extractable nuclear antigen (ENA) panel, anti-double-stranded DNA antibodies, anticardiolipin antibodies, anti-β2-glycoprotein antibodies, lupus anticoagulant, complement levels, rheumatoid factor, and muscle enzymes (creatine kinase, aldolase, aspartate aminotransferase, and lactate dehydrogenase), revealed no abnormalities. Serological testing for infectious agents (hepatitis B and C, parvovirus B19, Epstein–Barr virus, cytomegalovirus, *Mycoplasma pneumoniae*, *Borrelia* spp., *Toxoplasma* spp., and antistreptolysin O titer) was negative. Fungal infection was excluded by direct microscopy and culture. Patch testing with a standard allergen series showed no evidence of contact hypersensitivity. Extensive diagnostic workup did not identify any triggering factors or underlying infectious, neoplastic, neurologic, or autoimmune disease.

During further evaluation, a skin biopsy of the lesion on the right flank was performed, followed by histopathological and immunohistochemical examination.

Histopathological examination was performed on formalin-fixed, paraffin-embedded skin tissue obtained from the active border of the lesion [19,20]. Tissue was fixed in 10% neutral buffered formalin, dehydrated through graded ethanol solutions, cleared in xylene, and embedded in paraffin wax according to standard histopathological protocols. Serial sections of 3–4 µm thickness were cut and stained with hematoxylin and eosin (H&E) [19,20].

**Figure 2:**
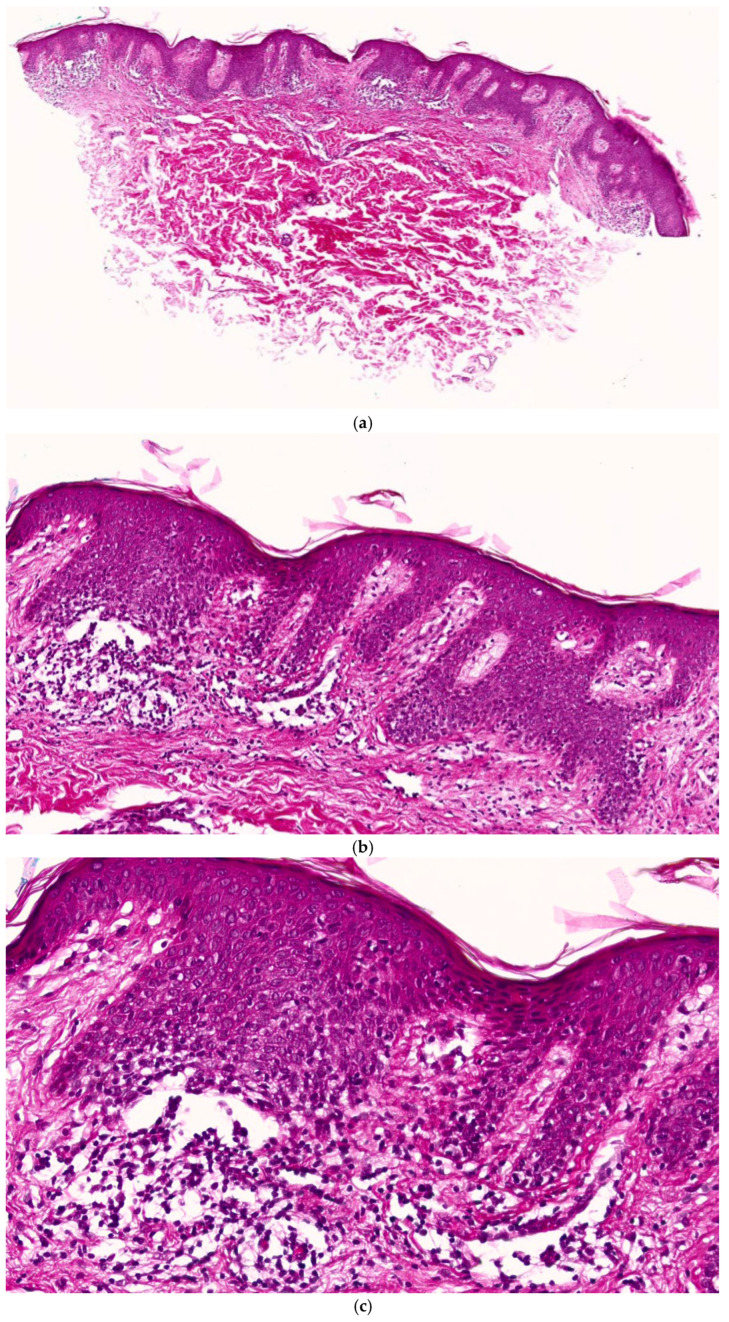
(**a**) Hematoxylin and eosin (H&E, ×10). Histopathological features of the lesion from the right flank. Hematoxylin and eosin-stained sections show regular epidermal acanthosis with elongation and partial fusion of adjacent rete ridges, resulting in a squared-off (quadrangular) configuration. (**b**) Hematoxylin and eosin (H&E, ×20). The infiltrate is predominantly confined to the tips of the rete ridges, with mild lymphocytic exocytosis and papillary dermal edema. Hematoxylin and eosin (H&E, ×40). (**c**) Lichenoid lymphocytic infiltrate is present at the dermoepidermal junction, accompanied by vacuolar (hydropic) degeneration of basal keratinocytes and scattered necrotic keratinocytes (Civatte bodies).

Microscopic examination revealed a mild to moderate hyperorthokeratotic stratum corneum without parakeratosis (Figure 2a–c). The epidermis showed regular acanthosis with elongation and partial fusion of adjacent rete ridges, resulting in broad, quadrangular (“squared-off”) rete ridges, a characteristic feature of annular lichenoid dermatitis of youth. Focal mild spongiosis was present (Figure 2a–c).

At the dermoepidermal junction, a lichenoid interface dermatitis was observed, characterized by vacuolar (hydropic) degeneration of basal keratinocytes, scattered necrotic keratinocytes (Civatte bodies), and focal lymphocytic exocytosis. The interface reaction was predominantly confined to the tips of the rete ridges (Figure 2a–c).

The papillary dermis demonstrated a superficial band-like inflammatory infiltrate composed predominantly of small mature lymphocytes, arranged in a lichenoid pattern. Occasional mast cells were present, while eosinophils were absent, and there was no evidence of neutrophilic infiltration or plasma cells. Mild papillary dermal edema was noted, without fibrosis (Figure 2a–c).

The inflammatory infiltrate extended focally along superficial adnexal structures but without follicular destruction. No significant cytologic atypia of lymphocytes, epidermotropism suggestive of mycosis fungoides, or dermal fibrosis was identified. The overall histopathological findings were consistent with annular lichenoid dermatitis of youth (Figure 2a–c).

**Figure 3:**
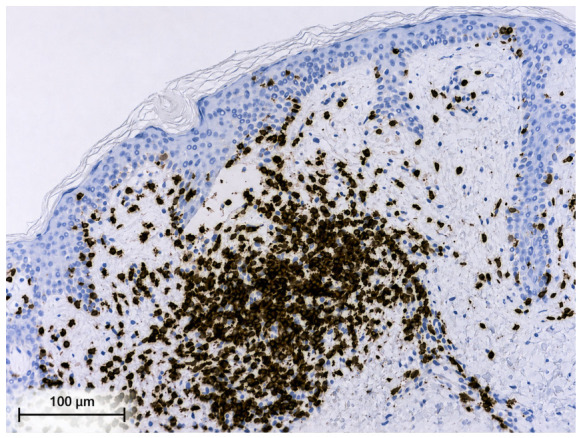
Immunohistochemistry revealed a pan-T-helper phenotype (CD3+ positive).

**Figure 4:**
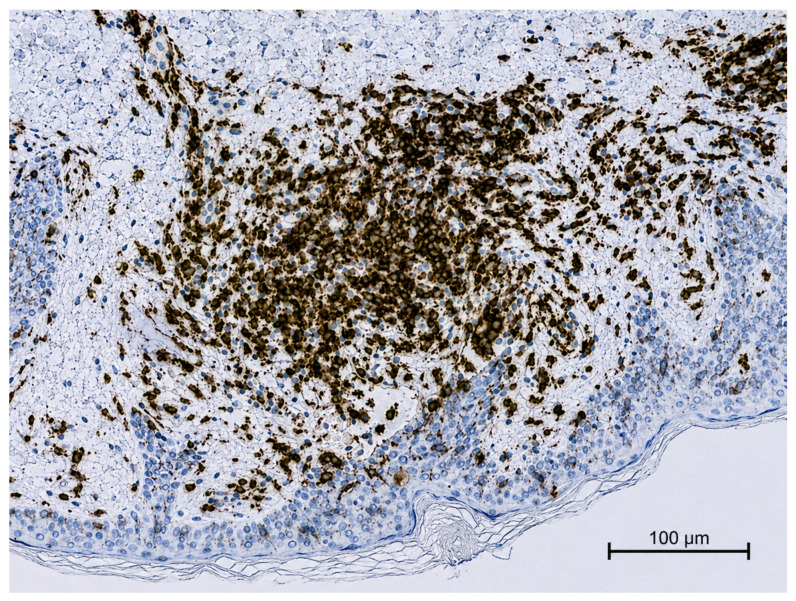
Immunohistochemistry revealed a pan-T-helper phenotype (CD4+ positive).

**Figure 5:**
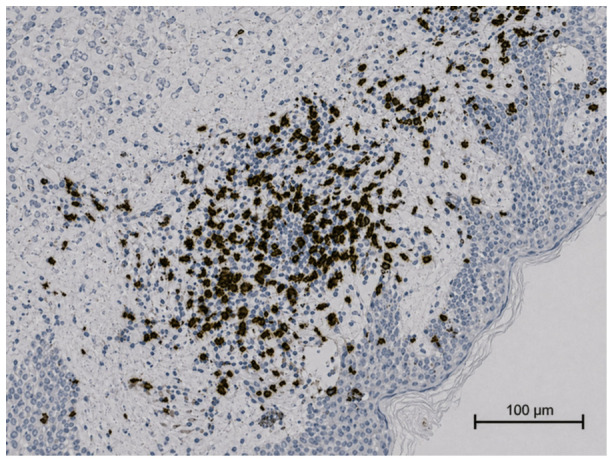
Immunohistochemistry: CD8 highlights a small number of T lymphocytes.

Immunohistochemical analysis revealed that the lymphocytes within the infiltrate exhibited a pan-T-helper phenotype (CD2+, CD3+, CD4+, CD5+, and CD7+). CD8 highlighted only a small number of T lymphocytes, and CD30 was negative (Figure 3, Figure 4 and Figure 5).

To assess T-cell clonality, molecular analysis of TCR gene rearrangements was performed on DNA extracted from skin biopsy specimens. T-cell clonality in patient samples was evaluated by PCR-based TCR genotyping, a standard method for detecting clonal rearrangements in TCR genes. Specifically, we used the BIOMED-2 clonality assay—ABI Fluorescence Detection (IdentiClone, InVivo Scribe Technologies, San Diego, CA, USA) according to the manufacturer’s instructions. DNA from fresh skin biopsies and peripheral blood was isolated using the High Pure PCR Template Preparation Kit (Roche Applied Science, Penzberg, Germany) according to the manufacturer’s protocol [21]. DNA concentration was measured using a NanoDrop spectrophotometer (Thermo Scientific, Wilmington, NC, USA) or a Qubit 3.0 fluorometer (Thermo Fisher Scientific, Waltham, MA, USA) [21].

T-cell clonality was evaluated using the IdentiClone TCRB + TCRG T-Cell Clonality Assay (InvivoScribe Technologies, San Diego, CA, USA), which enables detection of clonal rearrangements in the T-cell receptor β (TCRB) and γ (TCRG) genes [22]. The assay consists of three multiplex primer mixes for TCRB and two for TCRG, each containing fluorescently labeled primers. In addition, a control PCR targeting a housekeeping gene (Specimen Control Size Ladder) was performed to assess DNA integrity. DNA quality was considered sufficient when control PCR fragments of ≥400 bp were detected, or ≥300 bp in samples derived from formalin-fixed paraffin-embedded (FFPE) tissue. Each assay run included monoclonal and polyclonal control DNAs supplied with the BIOMED-2 assay, as well as a no-template control to monitor potential contamination [22].

PCR products labeled with fluorescent primers were analyzed by capillary electrophoresis on an ABI 3500 Genetic Analyzer (Applied Biosystems, Foster City, CA, USA) using fragment analysis. Data interpretation was performed according to the manufacturer’s recommendations and the EuroClonality/BIOMED-2 guidelines [22].

Monoclonal TCRB and TCRG gene rearrangements were detected in skin biopsy specimens. The lengths of the amplified products were consistent across the samples, with one exception: the monoclonal TCRB-A product (detected with the TCRB-A primer mix) was present only in the first skin biopsy and was not detected in the subsequent biopsies. In contrast, circulating lymphocytes in peripheral blood showed a polyclonal profile.

The patient was treated with several courses of topical corticosteroid ointment, which induced only transient regression of the lesion. Two years after the initial diagnosis, spontaneous resolution of the lesion was observed. After four asymptomatic years, the disease relapsed with identical clinical features.

During a lesion-free period, the patient reported no significant infections and had no previously diagnosed systemic diseases. Following recurrence of the skin lesions, additional investigations were repeated and revealed findings consistent with the previous results, including histopathological and immunohistochemical analyses. Molecular analysis of the skin again demonstrated a monoclonal T-cell clone, while circulating lymphocytes remained polyclonal.

## 3. Discussion

ALDY is an underrecognized dermatosis of unknown etiopathogenesis. Immunohistochemical findings suggest a cytotoxic T-cell-mediated immune reaction, similar to other lichenoid dermatoses [2,23]. With the exception of a single case reported after hepatitis B vaccination, no consistent associations with drugs, autoimmune diseases, neoplasms, ultraviolet exposure, infections, or immunologic alterations have been established [1,2,3,4,5,11,15]. Similarly, no triggering factors were identified in our patient. Serologic testing for *Borrelia burgdorferi* has been negative in all reported cases, including ours, and although Borrelia-like organisms have been detected in some lesions, a definitive association has not been confirmed [2,8,13].

The majority of reported ALDY patients have no relevant medical history; however, a subset has been associated with atopic conditions, including atopic dermatitis, asthma, allergic rhinitis, or celiac disease [1,2,3]. A similar atopic background was present in our patient.

To our knowledge, this is the first reported case of ALDY showing a monoclonal T-cell profile in both primary and recurrent skin lesions. This finding adds further complexity to the already challenging clinical and histopathological differentiation between ALDY and its main differential diagnosis, mycosis fungoides (MF).

The TCRG gene is characterized by a relatively restricted germline repertoire and limited junctional diversity within the rearranged Vγ–Jγ region, which may increase the likelihood of pseudoclonal results, particularly in samples with low T-cell numbers [24,25,26]. Although this raises the possibility of pseudoclonality in skin biopsies, the TCRG fragments identified in our patient were of identical length across all three biopsies, arguing against this phenomenon. Moreover, monoclonality was confirmed by TCRB clonality analysis, supporting the presence of a true monoclonal T-cell population within the lesional skin. In contrast, peripheral blood lymphocytes exhibited a polyclonal pattern, indicating that the clonal population is confined to the skin.

In addition, the presence of clusters of necrotic keratinocytes localized to the tips of the rete ridges, as observed in our case, represents a key histopathological feature favoring ALDY over mycosis fungoides (MF) [1].

In diagnostically challenging cases, analysis of T-cell receptor gene rearrangements represents a valuable adjunctive method for distinguishing ALDY from MF [1,2,7]. Notably, our patient demonstrated a monoclonal T-cell population, a finding not previously reported in ALDY.

Similar clinical presentations in adults are frequently associated with detectable T-cell clones, raising the suspicion of localized cutaneous T-cell lymphoma, particularly MF. However, the presence of a clonal T-cell population does not in itself establish a diagnosis of lymphoma. Conversely, a polyclonal pattern supports a diagnosis of ALDY [1,2,4,7,11,15], although it does not definitively exclude MF. In addition, cases of annular MF clinically mimicking ALDY have been described in both pediatric and adult patients [27,28].

Nevertheless, current evidence supports the view that ALDY represents a distinct clinicopathological entity, separate from MF [2,3], a distinction that may be particularly relevant in pediatric patients. In light of this diagnostic challenge, Stojkovic-Filipovic et al. recently proposed clinical, immunohistopathological, and molecular criteria for the diagnosis of ALDY [16], all of which were met in our patient.

The detection of monoclonal T-cell receptor rearrangement in our case raises the possibility of overlap with, or potential progression to, MF, as T-cell clonality can be identified in 52–75% of patch/plaque-stage MF lesions [7]. However, based on currently available evidence, it appears more likely that ALDY with monoclonal T-cell rearrangement represents a distinct T-cell lymphoproliferative process rather than classical MF.

The aim of this report is to highlight this unusual finding and to propose a potential novel entity, termed monoclonal annular lichenoid dermatitis of youth (MALDY). Given its clinical and histopathological overlap with other dermatoses, particularly MF, careful long-term follow-up and, when indicated, repeat biopsies during disease recurrence are essential.

Finally, we want to point out the potential limitations and recommendations. Our case report describes a single patient, and therefore the findings cannot be generalized. In addition, long-term follow-up data are lacking, which limits the ability to assess the clinical course and potential progression of the condition.

Despite these limitations, the rarity of ALDY and the novel molecular finding observed in our patient underscore the importance of further investigation. Additional case reports and larger studies are needed to clarify the clinical significance of T-cell clonality in this condition. Extended long-term clinical follow-up, together with repeated histopathological and molecular analyses in recurrent lesions, will be essential to determine whether MALDY represents a distinct benign inflammatory disorder or a localized T-cell lymphoproliferative disease within the spectrum of cutaneous T-cell disorders. This case also highlights the importance of considering this entity in similar clinical presentations, which may contribute to improved recognition and management.

## 4. Conclusions

In conclusion, we report a unique case of annular lichenoid dermatitis of youth characterized by monoclonal T-cell receptor gene rearrangement identified in both primary and recurrent lesions. To our knowledge, such a finding has not been previously described, as reported cases of ALDY have consistently demonstrated polyclonal T-cell populations. Although T-cell clonality is commonly associated with cutaneous T-cell lymphoma, it may also occur in benign inflammatory dermatoses. In the present case, the characteristic clinical and histopathological features, together with the absence of cytologic atypia, supported the diagnosis of ALDY.

These findings expand the current understanding of the molecular spectrum of ALDY and suggest the possibility of a distinct clinicopathological variant. We therefore propose the term monoclonal annular lichenoid dermatitis of youth (MALDY) as a tentative designation for this presentation. Recognition of this variant is important to avoid misdiagnosis as cutaneous T-cell lymphoma and to ensure appropriate clinical management.

Given the rarity of ALDY and the novel molecular finding described, further studies, additional case reports, and long-term follow-up are required to clarify the biological and clinical significance of T-cell clonality in this condition. Such investigations will be essential to determine whether MALDY represents a distinct benign inflammatory entity or a localized T-cell lymphoproliferative process within the spectrum of cutaneous T-cell disorders.

## Figures and Tables

**Table 1:** Overview of published literature on annular lichenoid dermatitis of youth (ALDY).

First Author (Year)	Study Type	No. of Patients	Age (Range)	Key Clinical Features	Key Histopathological Features	Treatment	Outcome
Annessi (2003) [1]	Case series	23	Children/adolescents	Annular erythematous patches, mainly trunk	Lichenoid infiltrate, basal vacuolar alteration	Topical corticosteroids	Generally benign, variable course
Sans (2008) [5]	Case report	1	Child	Annular plaques	Lichenoid dermatitis	Not reported	Not reported
Kleikamp (2008) [6]	Case report	1	12 y	Annular lesions	Lichenoid pattern	Tacrolimus	Good response
Cesinaro (2009) [7]	Case series	6	Children–adults	Annular lesions	Lichenoid/interface dermatitis	Topical/systemic therapy	Variable
Leger (2013) [8]	Case report	1	Child	Trunk involvement	Lichenoid infiltrate	Not reported	Not reported
Di Mercurio (2015) [9]	Case series	6	Children	Typical annular lesions	Lichenoid dermatitis	Topical corticosteroids	Good response
Kazlouskaya (2015) [10]	Case report	1	7 y	Annular patches	Lichenoid, MF-like features	Not reported	Stable
Vázquez-Osorio (2016) [11]	Case series	2	Children	Annular patches	Lichenoid infiltrate	Not reported	Not reported
Malachowski (2016) [12]	Case report	1	Child	Chronic relapsing lesions	Lichenoid dermatitis	Pimecrolimus	Good control
Wilk (2017) [13]	Case series	Several	Mixed	Annular lesions	Lichenoid, possible Borrelia association	Antibiotics (selected cases)	Variable
Cesinaro (2017) [14]	Case report	1	50 y	Atypical adult presentation	Lichen planus-like lichenoid	Not reported	Not reported
Debois (2018) [15]	Case report	1	Child	Annular plaques	Lichenoid dermatitis	Not reported	Not reported
Stojkovic-Filipovic (2020) [16]	Case report	1	Young	Recurrent disease	Lichenoid dermatitis	Cyclosporine	Good response
Bërdica (2025) [4]	Case report	1	Child	Annular lesion	Lichenoid dermatitis (diagnostic emphasis)	Not reported	Not reported

**Table 2:** Differential diagnosis of ALDY.

Condition	Clinical Features	Histopathology	Immunohistochemistry/Molecular Findings	Key Distinguishing Features vs. ALDY
ALDY	Annular erythematous-brown patches with hypopigmented center; asymptomatic or mild pruritus; trunk/flexures	Lichenoid/interface dermatitis localized to tips of rete ridges; keratinocyte necrosis; squared-off rete ridges	Predominantly CD3+, CD4+ dermal infiltrate; intraepidermal CD8+ cells; typically polyclonal TCR	Characteristic localization of interface dermatitis; benign course; usually polyclonal
Inflammatory morphea	Indurated plaques with violaceous border (“lilac ring”); may become sclerotic	Dermal sclerosis; thickened collagen bundles; reduced adnexal structures; mild inflammation	No specific clonality; inflammatory infiltrate variable	Presence of sclerosis and induration; absence of lichenoid pattern
Mycosis fungoides (MF)	Patches/plaques, often persistent, may resemble eczema; pruritus common	Epidermotropism of atypical lymphocytes; Pautrier microabscesses; atypia	Clonal TCR rearrangement common; CD4+ predominant	Cytologic atypia, epidermotropism, and clonal T-cell population; progressive course
Annular erythema	Annular erythematous lesions with centrifugal spread; often transient	Superficial perivascular infiltrate; minimal interface changes	No clonality	Lack of lichenoid/interface dermatitis; transient nature
Interstitial granulomatous dermatitis	Erythematous plaques, cords, or annular lesions; often associated with systemic disease	Interstitial histiocytic infiltrate; collagen degeneration; granulomatous features	No T-cell clonality	Presence of granulomatous inflammation and histiocytes

## Data Availability

The data supporting these case report findings are available from the corresponding author upon request.

## Abbreviations

The following abbreviations are used in this manuscript:

ALDY	Annular lichenoid dermatitis of youth
MF	Mycosis fungoides
MALDY	Monoclonal annular lichenoid dermatitis of youth
TCRB	T-cell receptor beta gene
TCRG	T-cell receptor gamma gene
tTG	Anti-tissue transglutaminase antibodies
EMA	Anti-endomysial antibodies
ENA	Extractable nuclear antigen
PCR	Polymerase chain reaction
DNA	Deoxyribonucleic acid
TCRB + TCRG T-Cell Clonality Assay	Molecular assay for detection of T-cell clonality using T-cell receptor beta and gamma gene rearrangements
H&E	Hematoxylin and eosin
